# An ECM-Mimicking, Mesenchymal Stem Cell-Embedded Hybrid Scaffold for Bone Regeneration

**DOI:** 10.1155/2017/8591073

**Published:** 2017-11-15

**Authors:** Jozafina Haj, Tharwat Haj Khalil, Mizied Falah, Eyal Zussman, Samer Srouji

**Affiliations:** ^1^Faculty of Medicine, Technion, 3109601 Haifa, Israel; ^2^Institute of Oral and Maxillofacial Surgery and Oral Medicine, Galilee Medical Center, 2210001 Nahariya, Israel; ^3^Research Center, Galilee Medical Center, 2210001 Nahariya, Israel; ^4^Faculty of Medicine in the Galilee, Bar-Ilan University, 1311502 Safed, Israel; ^5^Faculty of Mechanical Engineering, Technion, 3200003 Haifa, Israel

## Abstract

While biologically feasible, bone repair is often inadequate, particularly in cases of large defects. The search for effective bone regeneration strategies has led to the emergence of bone tissue engineering (TE) techniques. When integrating electrospinning techniques, scaffolds featuring randomly oriented or aligned fibers, characteristic of the extracellular matrix (ECM), can be fabricated. In parallel, mesenchymal stem cells (MSCs), which are capable of both self-renewing and differentiating into numerous tissue types, have been suggested to be a suitable option for cell-based tissue engineering therapies. This work aimed to create a novel biocompatible hybrid scaffold composed of electrospun polymeric nanofibers combined with osteoconductive ceramics, loaded with human MSCs, to yield a tissue-like construct to promote* in vivo* bone formation. Characterization of the cell-embedded scaffolds demonstrated their resemblance to bone tissue extracellular matrix, on both micro- and nanoscales and MSC viability and integration within the electrospun nanofibers. Subcutaneous implantation of the cell-embedded scaffolds in the dorsal side of mice led to new bone, muscle, adipose, and connective tissue formation within 8 weeks. This hybrid scaffold may represent a step forward in the pursuit of advanced bone tissue engineering scaffolds.

## 1. Introduction

Bone regeneration is a complex physiological process, which occurs continuously during adult life, as well as during normal fracture healing. However, there are complex clinical situations, such as bone loss due to trauma, infection, or disease, in which large quantities of bone regeneration are required [1, 2]. Currently, there are several clinical approaches to address insufficient bone repair and regeneration, including bone grafting techniques which apply autografts, allografts, and alloplastic bone grafts [2]. Although the current treatment strategies have been shown to improve bone repair and are commonplace in orthopedic surgery, none features the full gamut of ideal characteristics, such as high osteoinductive and angiogenic potentials, biological safety, low patient morbidity, scalability, extended shelf-life, and cost-effectiveness [3]. For example, using the current strategies, it is difficult to obtain the quantities of tissue necessary to replace large bone defects. Bone tissue engineering (BTE) has evolved to fill this unmet need [1, 2]. The classic BTE process involves (1) a 3-dimensional (3D) scaffold that mimics the natural bone extracellular matrix niche, (2) osteogenic cells, which deposit bone tissue matrix, (3) morphogenic signals, which trigger differentiation of the osteogenic cells to the phenotypically desirable cell type, and (4) sufficient vascularization to meet the growing tissue nutrient supply and clearance demands [3].

Over the past few decades, numerous biomaterials have been proposed as “ideal” for cell growth on implantable grafts, yet few have demonstrated clinical efficacy. In the case of bone regeneration, the materials must demonstrate biocompatibility, osteoinductivity, to promote osteoblastic differentiation, osteoconductivity, to support new bone growth, osteointegrativity, to provide biological fixation of scaffold to bone, angiogenesis, to ensure long-term functionality of the graft, and mechanical compatibility with native bone [4–6].

Generally, the scaffold is comprised of polymers, ceramics, or a composite of the two, depending on the intended application of the scaffold [5, 7]. Polymers display a range of physical and mechanical properties, degradation times, and modes, and they have vast design flexibility, allowing for tailoring of graft composition and structure to specific needs. In contrast, ceramics, which are formed from inorganic, nonmetallic materials that can take on a crystalline structure, are ideal scaffolding candidates as the inorganic component of bone, due to its close resemblance with native apatite of the human skeleton. Ceramics that are composed of hydroxyapatite have the ability to chemically bind live bone tissue and to enable osteoblast adhesion and proliferation [7, 8]. However, they are nondegradable in a biological environment and display limited processability. Therefore, they are disadvantageous for tissue engineering applications [7, 9]. Composite polymer and ceramic materials can significantly synergize with each other to reduce the overall brittleness of ceramics and to increase the porosity, bioactivity, and the osteoconductivity of the polymeric scaffold [7].

Some of the most promising research efforts in the field of regenerative medicine have focused on the use of stem cells, which display both self-renewing and broad differentiation capacities [10, 11], alongside accessibility and expansibility. Mesenchymal stem cells (MSCs) comprise a subtype of multipotent stem cells, and they are highly sought after in research due to their ease of isolation [12].

This study examined the potential of a 3D multilayered, hybrid scaffold composed of osteoconductive ceramic particles and polymeric polycaprolactone (PCL) nanofibers, to support human MSC proliferation and differentiation into bone tissue when subcutaneously implanted into an ectopic mouse model.

## 2. Materials and Methods

### 2.1. Fabrication of Cell-Embedded Electrospun Scaffolds

The osteoconductive ceramic particles used in this study are Pro Osteon 200R. Pro Osteons (full 200 microns) are coralline-derived resorbable, osteoconductive particles consisting of a thin 2–10 *μ*m layer of hydroxyapatite (HA) over a calcium carbonate core. Pro Osteons provide continuous pathways for bony ingrowth through their interconnected porosity and are consisted of small granules (0.5–1 mm), which make them convenient for filling small defects. Their architecture and chemical composition are similar to human bicortical bone.

**Figure 1:**
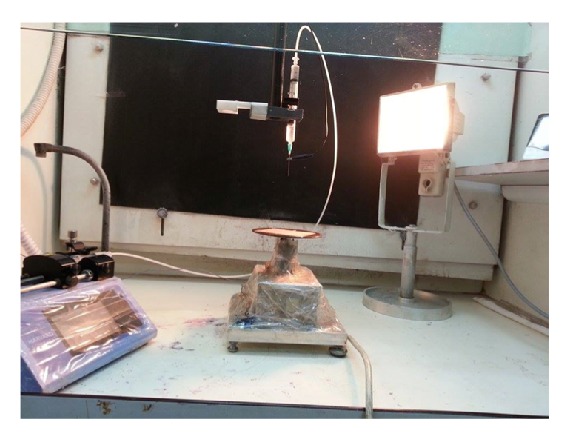
*Apparatus for fabrication process of the hybrid scaffold.* An electrospinning apparatus was built consisting of syringe pump, high-voltage power supply, and rotated collector.

To produce a hybrid scaffold of PCL nanofibers and Pro Osteon particles, an electrospinning apparatus was built consisting of syringe pump, high-voltage power supply, and rotated collector (Figure 1). The nanofibers were electrospun from an a 9% w/v solution of PCL (Mw 80,000 Da; Sigma Aldrich) dissolved in a 9 : 1 mixture of chloroform (CHCl_3_) and dimethyl sulfoxide (DMSO). The electrospinning solution was ejected vertically from a plastic syringe outfitted with a 23-gauge blunt tipped needle at a flow rate of 3 ml/h for 15 min, at an applied voltage of 12 kV. To create mat of the hybrid scaffold, rotated flat aluminum collector was located 10 cm below the needle tip. A mat (9 cm^2^) of hybrid scaffold is composed of 10 layers of PCL fibers (750 *μ*l), scattered between them is 150 mg of ceramic particles (ratio 5 : 1). This ratio was determined based on our previous studies that optimize the suitable ratio for highest scaffold porosity.

Prior to cell seeding, scaffold samples were sterilized by soaking them overnight in 70% ethanol and then washed several times with phosphate buffered saline (PBS).

### 2.2. Cell Isolation and Culture Conditions

The study was approved by the Rambam Health Care Campus Helsinki Committee (#0370-12-‎RMB).

MSCs were isolated from human adipose tissue, according to a protocol established by Zeng et al. [13]. Human adipose tissue, extracted by liposuction, was cut into pieces and then allowed to adhere to the walls of culture plates. On the third day of culture, MSCs were released from the edges of adipose tissue. Cells were cultured in basic growth medium comprised of Dulbecco's Modified Eagle Medium (DMEM), 10% fetal bovine serum (FBS), 1% L-glutamine, and 1% penicillin-streptomycin (all purchased from Biological Industries) and then expanded up to passage 3 or 4 before being used in* in vitro* (1 × 10^6^ cells per scaffold) and* in vivo* studies (2 × 10^6^ cells per scaffold). MSCs were cultured in inductive conditions before being seeded on the scaffold. Inductive conditions were achieved by culturing MSCs in inductive medium, composed of DMEM, 10^−8 ^M dexamethasone, and 100 *μ*g/ml L-ascorbic acid 2-phosphate sesquimagnesium (all purchased from Sigma Aldrich) for 7 days. To verify the osteogenic potential of the isolated MSCs, their osteogenic differentiation was induced by culturing them for 28 days in osteoinductive medium, containing inductive medium supplemented with 10 mM *β*-glycerophosphate (Sigma Aldrich). Medium was changed every 3 days.

### 2.3. Scaffold Morphological Characterization

The morphology of the unseeded/seeded hybrid scaffold was characterized using scanning electron microscopy (SEM).

Unseeded hybrid scaffolds were sputter-coated with gold palladium while seeded scaffolds were fixed in 0.1 M NBF (24 h), followed by 1% OsO_4_ (1 h) and 2% tannic acid (1 h). They were then dehydrated in graded ethanol solutions, sputter-coated with gold palladium. All samples were photographed using a Phenom scanning electron microscope (PhenomWorld).

### 2.4. Porosity

To evaluate the relation between mass and porosity of the hybrid scaffolds, scaffold mass was manipulated by dispersing different amounts of ceramic particles and by that changing the ratio between them and the PCL nanofibers. Three samples were cut from each hybrid mat. While the sample was trapped between two glass plates, the thickness of each sample was measured with a digital caliper (accuracy of ±1 *μ*m). Sample weight was measured with a digital scale (accuracy of ±0.1 *μ*m).

The porosity, *Ø*, of each scaffold was calculated according to (1), where *ρ*_bulk_ is the measured scaffold density (determined from sample weight and volume, *ρ*_bulk_ = *m*/*v*) and *ρ*_particle_ is the standard PCL density (*ρ*_particle_ = 1.145 g/cm^3^).

(1) $$\begin{array}{c} Ø=1-\frac{\rho_{bulk}}{\rho_{particle}}. \end{array}$$

### 2.5. Permeability

Permeability tests were performed using a permeability rig, as previously described [14]. Permeability was determined by permeability constant-*k*, using Darcy's Law:

(2) $$\begin{array}{c} k=\frac{{Q\cdot \mu }_{air}}{A}\frac{\Delta L}{\Delta P}, \end{array}$$

where *k* is the intrinsic permeability (m^2^), *Q* is the air flow rate into the sample (m^3^/sec), *μ* is the air viscosity (Pa·sec), *A* is the sample's cross-sectional area (m^2^), Δ*L* is the thickness of the sample (m), and Δ*P* is the pressure difference between the sample lumen and the external atmosphere (Pa).

### 2.6. Cell Proliferation Assay

Cells (1 × 10^6^) were seeded on each scaffold and cultured in basic growth medium for 21 days. The adhesion of cells was determined one day after by washing the seeded scaffolds twice in PBS to remove unadhered cells and incubating them for 2 h in medium containing 10% Alamar Blue dye (Serotec). Fluorescence was recorded using a FLUOstar galaxy fluorescence reader, at an excitation wavelength of 540 nm and emission wavelength of 580 nm (BMG Labtech). The same procedures were performed to determine the proliferation rate of cells on the time points of 3, 7, 14, and 21 days.

### 2.7. Immunophenotyping of MSCs

Cultured MSCs were trypsinized, centrifuged, and transferred to flow cytometry tubes (1 × 10^5^ cells per tube) containing working buffer (PBS with 2% FBS) and then centrifuged at 1250 rpm for 10 min. After discarding the supernatant, the cells were incubated with mouse anti-human phycoerythrin- (PE-) conjugated antibodies: anti-CD45 (Bactlab Diagnostics), anti-CD73 (Bactlab Diagnostics), anti-CD34 (Bactlab Diagnostics), anti-CD90 (Biotest), anti-CD105 (Ornat), anti-CD11b (Bactlab Diagnostics), anti-CD19 (Bactlab Diagnostics), anti-HLA-DR (Biotest), or PE-conjugated anti-mouse IgG (Bactlab Diagnostics) for 15 min in the dark. Then, they were washed with the working buffer, centrifuged, fixed with neutral-buffered formalin (NBF), and analyzed with a BD LSRFortessa cell analyzer (BD Bioscience).

Marker expression was analyzed using FlowJo software (FlowJo) and is presented as the percentage of fluorescence-positive cells.

### 2.8. Cell Differentiation Assay

After 28 days of incubation in osteoinductive medium, cultured cells were rinsed twice in PBS, fixed in 0.1 M neutral-buffered formalin (NBF) (10 min), rinsed with PBS, and then stained with 2 ml of 2% alizarin red S solution (Sigma) for 0.5–5 min to identify calcium deposits, which are indicators of mature osteocytes.

Alternatively, cells were rinsed twice with PBS, fixed with 0.1 M NBF (2 min), rinsed with PBS, and stained with 2 ml alkaline phosphatase (ALP) solution (Sigma) for 30–50 min at 37°C. Alkaline phosphatase stains cells blue-violet if they contain active ALP. Staining was determined using an inverted light microscope (Olympus).

### 2.9. Scaffold Implantation* In Vivo*

Cells were trypsinized and counted and then resuspended in 50 *μ*l inductive medium before being seeded onto the scaffold (2 × 10^6^ cells per scaffold), according to our previously established studies [15, 16]. The cell-seeded scaffolds were then incubated for 70 min in the incubator, with slow rotation, and then soaked in inductive medium for one week. On the implantation day, scaffolds were stabilized with a fibrin clot coat, composed of 1 : 1 rat fibrinogen : rat thrombin (Sigma Aldrich).

All of the described surgical procedures were performed in accordance with protocols approved by the Institutional Animal Care and Use Committee (IL0810712). Five groups of 6-week-old, nude female mice (*n* = 5 per group, Harlan Laboratories) were anesthetized using a 0.5 : 0.5 : 9 ketamine : xylazine : PBS cocktail at a dose of 400 *μ*l/20 g body weight, delivered via a 25-gauge needle. Cell-seeded constructs were then subcutaneously implanted in the dorsal side of the mice. Acellular scaffolds and cell-seeded osteoconductive particles (Pro Osteon, 40 mg) that were also precoated with a fibrin clot were subcutaneously implanted as negative and positive controls, respectively. Tissue samples of the construct area were extracted for histological analysis 8 weeks after implantation.

### 2.10. Histological Analysis

MSC-embedded scaffolds* (in vitro)* and extracted MSC-embedded scaffolds* (in vivo)* were fixed in 0.1 M NBF for 24 h, followed by decalcification in ethylenediaminetetraacetic acid (EDTA) solution for one week, until no traces of calcified tissue remained. Specimens were then dehydrated in graded ethanol solutions (70%, 95%, and 100%), embedded in paraffin, and then cut with a microtome to 7 *μ*m thick histological sections. Finally, the samples were stained with hematoxylin and eosin (H&E), to identify the nucleus and the connective tissue. Histological slides were scanned using an automatic digital slide scanner (3D Histech Pannoramic MIDI, 3DHISTECH) and the percentage of the newly formed bone was quantified using the panoramic viewer software (3DHISTECH.).

### 2.11. Statistical Analysis

All experiments included 4 or 5 replicates. All data are expressed as mean ± standard error of mean. A two-tailed Student's *t*-test was performed to compare between two groups. *P* value < 0.05 was considered statistically significant. All statistical analyses were performed using the GraphPad Prism 5 software.

## 3. Results

### 3.1. Scaffold Design and Characterization

**Figure 2:**
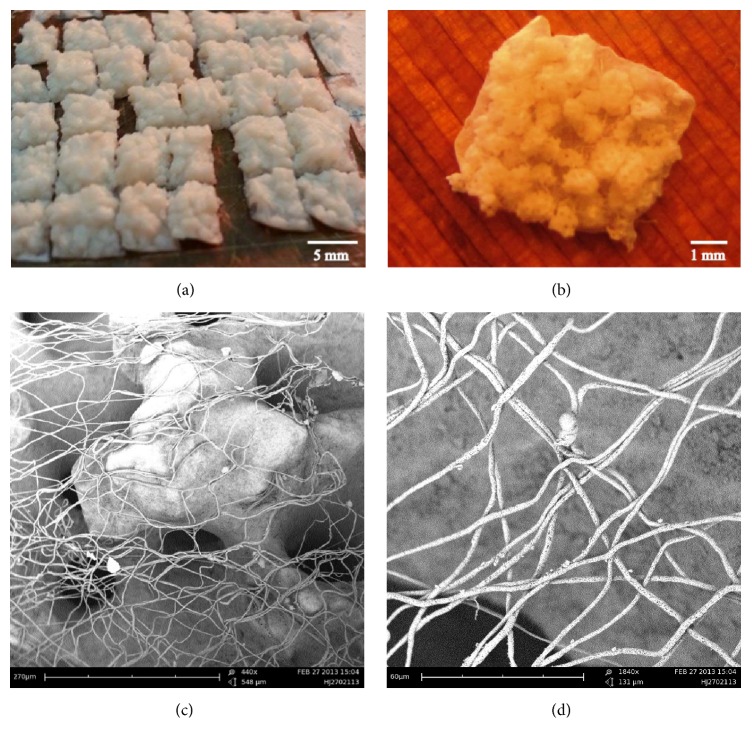
*Images of the electrospun composite scaffold.* (a) Mat of hybrid scaffolds. (b) Samples of an electrospun scaffold just before SEM and physical analyses. (c) PCL fibers and osteoconductive particles, as observed by SEM (×440). (d) SEM image of the electrospun scaffold fibers (×1840).

Initial research efforts focused on designing an adequate ECM-like scaffold model prepared from electrospun polymeric nanofibers and osteoconductive ceramics. The hybrid electrospun scaffolds were up to ~125 mm^3^ and had an average thickness of 235 ± 22 *μ*m (Figures 2(a) and 2(b)). SEM images (Figures 2(c) and 2(d)) revealed the 3D meshed structure of the hybrid scaffold, comprised of multilayers of PCL fibers and osteoconductive particles dispersed throughout. This combination resembled the nano- and microarchitecture of bone ECM, with the PCL fibers and the Pro Osteon particles mimicking the collagen fibers and the calcium/phosphate (hydroxyapatite) bone ECM components, respectively.

**Figure 3:**
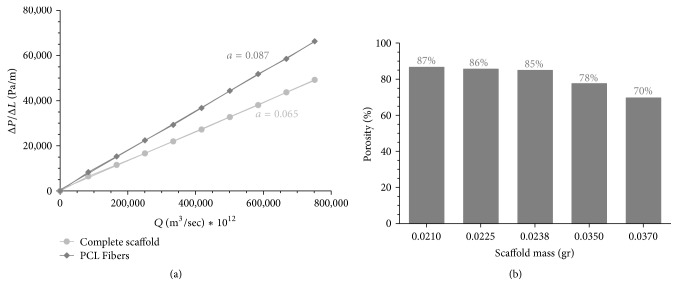
*Physical characteristics of the electrospun scaffold and PCL-only scaffold. *(a) Permeability of hybrid scaffold versus PCL-only scaffolds was calculated using Darcy's law (see (2); *a* = *μ*_air_/(*A* · *k*)). (b) Scaffold porosity versus mass.

In order to examine the effect of combining Pro Osteon particles with the PCL fibers, two physical parameters—permeability and porosity—were measured. The relationship between the measured pressure/sample thickness and the air flow rate through the sample is depicted in Figure 3(a), showing that the permeability of the hybrid scaffolds was 1.5-fold higher than the ceramic-free scaffolds (14.565 versus 10.595 Darcy, resp.). Scaffold porosity proved mass-dependent, demonstrating that scaffolds with lower masses, as a result of dispersing more Pro Osteon particles and changing the ratio of PCL nanofibers and Pro Osteon particles, exhibited higher porosity, indicating a positive effect of Pro Osteon particles on porosity (Figure 3(b)). Therefore, low-mass scaffolds were used in further assessments.

### 3.2. Morphological and Phenotypical Validation of the Isolated MSCs

**Figure 4:**
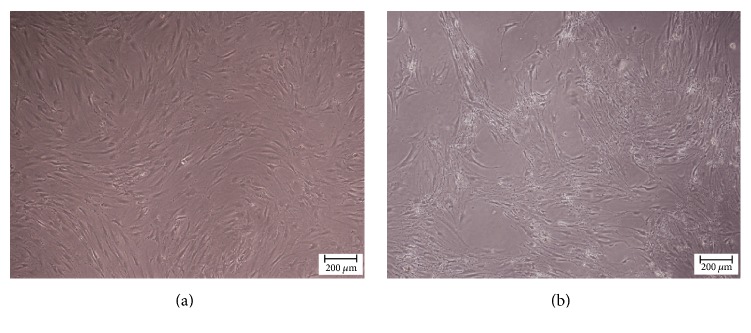
*Characterization of MSCs.* Isolated cells were cultured in basic or inductive medium and their morphology in culture was examined using an inverted microscope. (a) Unstained spindle-shaped cells after 7 days of culture in basic medium. (b) Unstained cell clusters after 28 days of culture in inductive medium (scale bar: 200 *μ*m).

**Figure 5:**
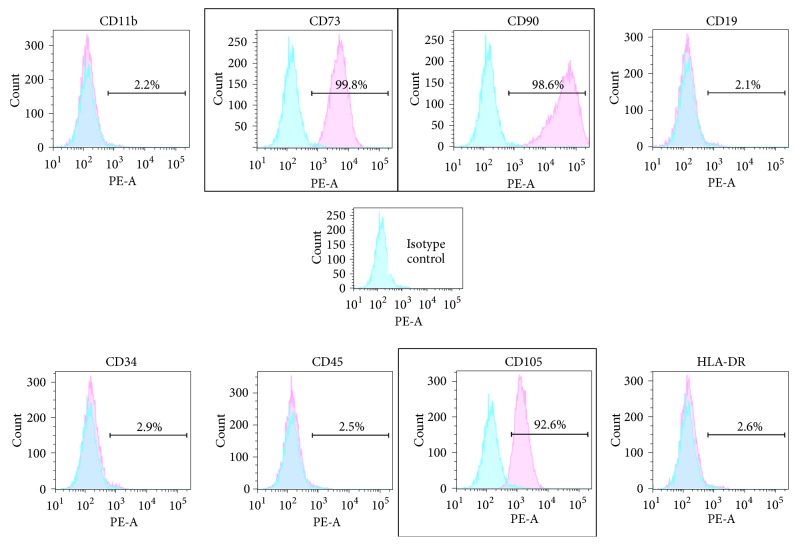
*Flow cytometry analysis of MSCs cultures. *Adipose-derived MSCs were cultured for 28 days in basic medium, trypsinized, and stained with fluorescent antibodies to mesenchyme-specific markers (CD73, CD105, and CD90; framed), hematopoietic markers (CD34, CD45), and leukocyte markers (CD11b, CD19, and HLA-DR) and analyzed using flow cytometer to confirm their MSC phenotype.

Before seeding the MSCs on the hybrid scaffold, their morphological and phenotypical characterization was verified. Human MSC morphology resembled that of fibroblasts, with a distinct spindle-like shape that was retained for up to 7 days in basic growth medium (Figure 4(a)). Following culturing of up to 28 days in inductive conditions, the cells began to form clusters (Figure 4(b)). Flow cytometry analysis verified MSC identity, with the expected expression of CD73, CD90, and CD105 (99.8%, 98.6%, and 92.6% of cell population, resp.), and absence (<3%) of the hematopoietic and the leukocyte markers CD34, CD45, CD11b, CD19, and HLA-DR (Figure 5).

**Figure 6:**
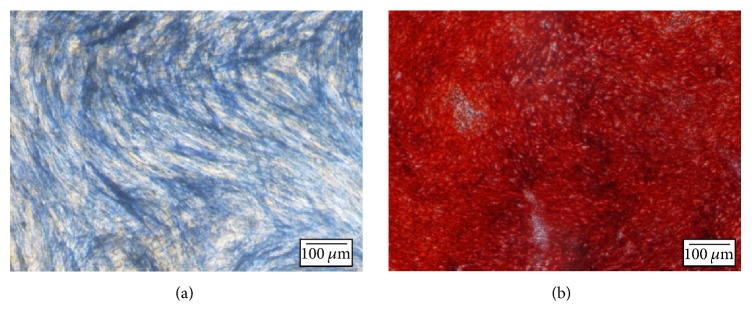
*Validation of osteogenic potential of MSCs.* MSCs were cultured in osteoinductive medium for 28 days and then stained with alkaline phosphatase and alizarin red to evaluate their osteogenic differentiation. (a) Alkaline phosphatase staining was used to detect active ALP. (b) Alizarin red staining was used to detect calcium deposits.

The osteogenic differentiation potential of the isolated MSCs, which were cultured in osteogenic conditions in culture plate, was demonstrated by expression of both early and late osteogenic markers (ALP and calcium) (Figures 6(a) and 6(b), resp.).

### 3.3. MSC Expansion and Attachment to the Hybrid Scaffold

**Figure 7:**
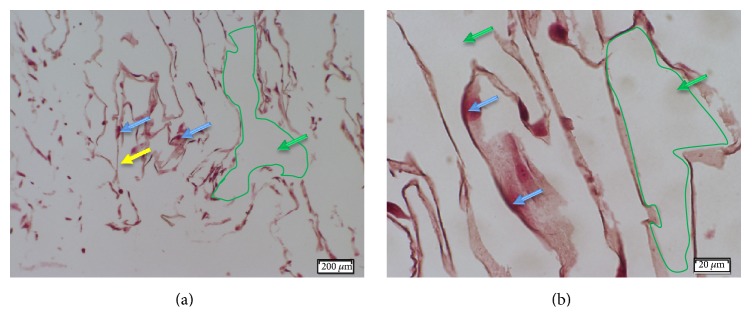
*H&E staining of MSC-embedded hybrid scaffolds.* MSC-embedded hybrid scaffolds were cultured in inductive medium for 7 days. Then, they were fixed, demineralized, and embedded in paraffin, and histological sections were stained with H&E. MSCs were extensively expanded within the hybrid scaffold: (a) ×40 and (b) ×400. PCL fibers and cell nuclei are indicated by yellow and blue arrows, respectively. Pro Osteon® particle positions before demineralization are bordered with green line and indicated with green arrows.

Histological analysis demonstrated extensive MSC expansion within the meshed structure of the hybrid scaffold, after 7 days of incubation in inductive medium (Figure 7).

**Figure 8:**
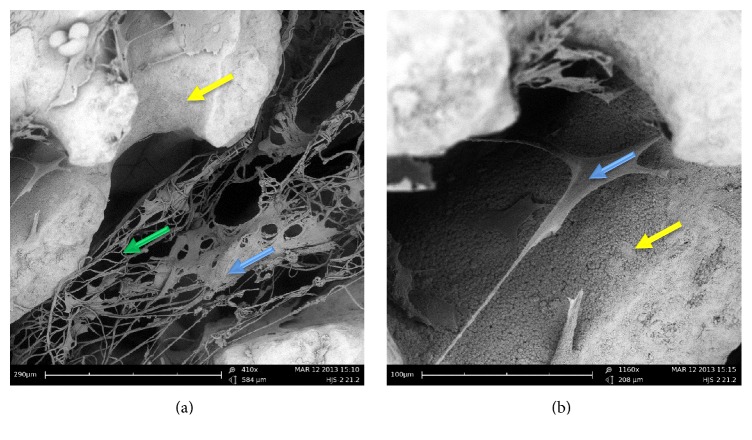
*SEM images of cell-embedded hybrid scaffolds. *MSC-embedded hybrid scaffolds were cultured in inductive medium for 7 days. Then, they were fixed, coated with gold, and analyzed using SEM. (a) MSC expansion on the PCL fibers (×410). (b) MSC adherence to Pro Osteon particles (×1160). MSCs, Pro Osteons, and PCL fibers are indicated by blue, yellow, and green arrows, respectively.

In contrast to histological sections, wherein the ceramics are dissolved through a demineralization process, SEM imaging allows for observation of the Pro Osteon particles. SEM images demonstrated MSC attachment (Figure 8, blue arrows) to the scaffold components, and their growth along the PCL fibers (Figure 8(a), green arrows) and the osteoconductive particles (Figure 8, yellow arrows). These observations confirmed the ability of the scaffold components in support of the seeded cells.

### 3.4. The Hybrid Scaffolds Supported MSCs Proliferation

**Figure 9:**
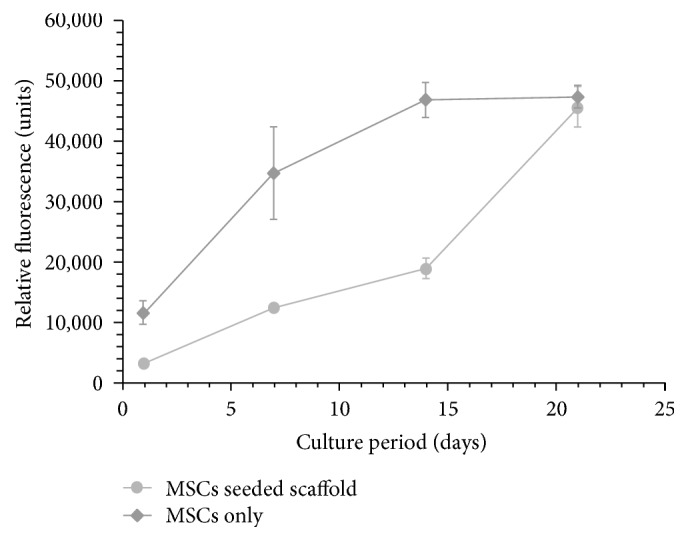
*MSC proliferation on hybrid scaffolds.* MSC-embedded hybrid scaffolds were cultured in basic growth medium for 21 days. MSC proliferation was determined on days 1, 3, 7, 14, and 21 days thereafter, using Alamar Blue assay, and was compared to that of MSCs cultured alone. Data are presented as mean ± standard error of mean of relative fluorescence of three triplicates of three experiments.

In order to assess whether the hybrid scaffolds provide adequate biological support for growing cells, the proliferation rate of seeded cells was monitored. Seeded cells demonstrated proliferative capacities which increased with culture time (Figure 9). Of note, the proliferation rate of the MSCs only, cultured on a tissue culture dish (control), was greater than that of the MSCs grown on scaffolds (Figure 9). This difference could be related to the favorable MSCs initial adherence to plastic flasks rather than to PCL fibers taking into consideration the 2D versus 3D scaffold characteristics of culturing. Cells adhere easily to 2D flasks, while when seeded onto scaffolds they should integrate and adhere before being washed. In addition, cells cultured onto 2D surfaces are exposed more to nutrients and oxygen and thus grow faster compared to cells cultured on 3D scaffold.

### 3.5. MSC-Seeded Scaffolds Implanted into Ectopic Mouse Model

**Figure 10:**
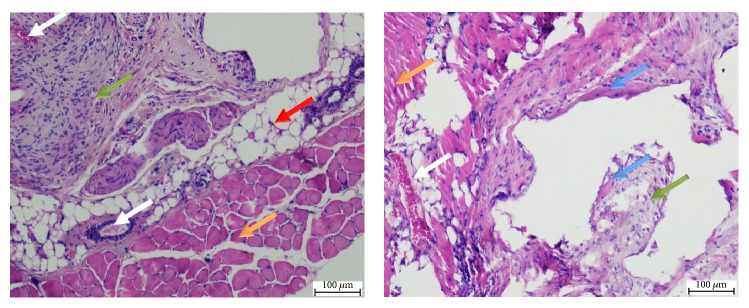
*H&E-stained histological sections of the cell-embedded hybrid scaffold subcutaneously implanted into mice. *Scaffolds were extracted 8 weeks after implantation and histological sections were stained with H&E. Several tissue types were identified: muscle tissue (orange arrows), blood vessels (white arrows), adipose tissue (red arrow), connective tissue (green arrows), and bone tissue (blue arrows).

In order to examine the biocompatibility of the MSC-seeded hybrid scaffolds, scaffolds were seeded with induced cells and then subcutaneously implanted in mice. Eight weeks after implantation, implants were extracted and samples were stained with H&E. Cells were well integrated within the implanted scaffolds (Figure 10) and succeeded in forming several tissue types in each scaffold, including muscle tissue, blood vessels, adipose tissue, connective tissue, and bone tissue, as identified by their histological structure.

**Figure 11:**
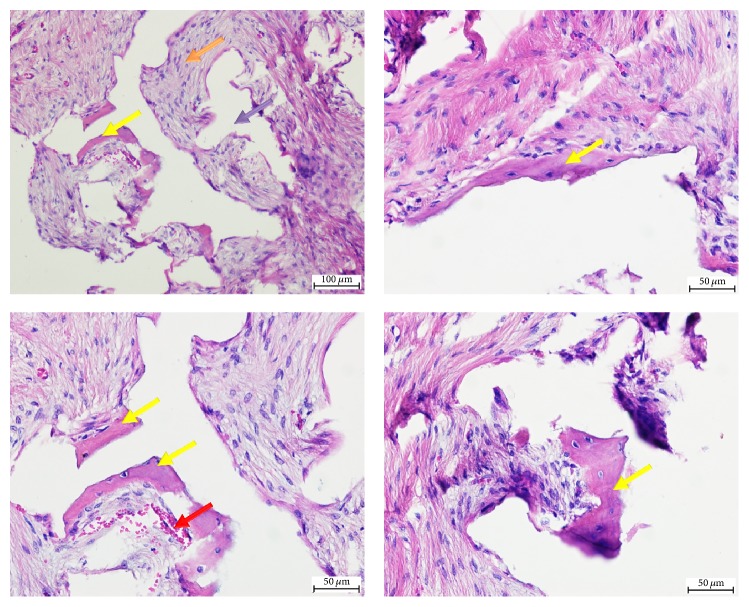
*Bone tissue formation in MSC-embedded hybrid scaffolds.* Subcutaneously implanted MSC-seeded hybrid scaffolds were extracted 8 weeks after implantation and histological sections were prepared and stained with H&E stains. Four sections of different fields demonstrate the newly formed bone tissue (yellow arrows), blood vessels (red arrow), connective tissue (orange arrow), and Pro Osteon particle positions before demineralization (purple arrow).

To determine the osteogenic potential of the hybrid scaffolds, scaffolds were subcutaneously implanted in the dorsal side of mice and tissue samples were extracted for histological analysis 8 weeks after implantation. New bone tissue formation was observed within the MSCs-seeded scaffolds (Figure 11; yellow arrows and margins), in addition to muscle, adipose, and connective tissues. No significant difference in bone area was measured between the hybrid scaffold and positive control samples treated with Pro Osteon particles seeded with MSCs (12.97% versus 11.35%, *P* = n.s.). In contrast, animals treated with acellular scaffolds showed muscle, adipose, and connective tissues but no bone tissue in the scaffold area (data not shown). Formation of these tissues in the negative controls was likely due to migration of mouse cells to the implanted scaffold.

## 4. Discussion

Tissues generated using bone tissue engineering strategies by combining biomaterials, cells, and signaling factors are seen as alternatives to conventional bone grafts for repairing or rebuilding bone defects [17]. The main challenge in producing a biocompatible and functional engineered tissue for bone regeneration is appropriate selection of the biomaterials, structure, and cell types. This is to obtain functional scaffold that mimics the biomechanical and biochemical properties of the natural tissue's extracellular matrix (ECM) [18]. Bone ECM is comprised of collagen fibers and hydroxyapatite, which is composed of calcium and phosphate [19, 20].

In this study, we present a novel bioengineered 3D scaffold that mimics the nano- and microstructure of bone ECM, designed to replace the collagen fibers and hydroxyapatite minerals with electrospun PCL nanofibers and osteoconductive Pro Osteon ceramics, respectively. The scaffold has a preliminary, simple, and flexible 3D design. In order to generate a 3D construct with a nano- and microarchitecture characteristic of the complex molecular architecture of bone ECM, the fabrication process combined electrospinning technology with the multilayer technique to produce multilayer-PCL nanofibers that resemble collagen fibers at the nanolevel. Such fibers were separated by layers of osteoconductive particles, resembling the ECM at the microlevel. Electrospinning was employed due to its superiority over other 3D biofabrication techniques in cost-effectiveness, simplicity, compatibility with a wide range of materials, high surface-to-volume ratio, and high flexibility in controlling fiber diameter and spatial orientation [21–23].

The scaffold porosity plays an important role in directing tissue formation and function and is often necessary to allow for homogeneous migration and distribution of cells throughout the interconnected engineered tissues [24]. A porous synthetic scaffold is thought to be needed to provide the necessary support for cells to proliferate and maintain their functions and to guide three-dimensional tissue regeneration [25–27]. The pores allow nutrient diffusion and waste removal to and from the regeneration site, especially in the absence of a functional vascular system, as well as providing appropriate mechanical environment to effectively support cell growth [24, 28]. The porosity of neat PCL scaffold, fabricated by different techniques, range from 27% to 77%, while the porosity of trabecular bone is higher [29–31]. Therefore, the hybrid scaffold was carefully designed to provide relevant porosity and permeability, both of which directly influence oxygen transport, nutrient supply, waste removal, and cell migration within the scaffold [32, 33]. According to SEM images, the presented technique produced 3D porous scaffolds with micro- and nanoarchitecture that support cell integration and expansion. Furthermore, the measured permeability and porosity of the hybrid scaffolds (1.437 × 10^−11 ^m^2^ and 85–87%, resp.) were within the range of those reported for human trabecular bone, confirming that the scaffold represents an artificial extracellular matrix structure relevant to bone tissue engineering applications [3, 33]. Moreover, Mitsak et al. demonstrated that high-permeability PCL scaffolds supported considerable* in vivo* bone ingrowth, which, in turn, increased the mechanical properties of PCL scaffolds [28]. In this study, we demonstrated that the permeability of the engineered hybrid scaffold, obtained due the combination of the electrospinning and multilayer techniques, was higher than PCL nanofibers alone.

Mimicking the nanotopography of natural ECM is advantageous for successful regeneration of damaged tissues or organs. Approaches that successfully reach nanoscale level resemblance of the physiological bone tissue environ may provide significant benefits in tissue regeneration processes. Of the nanostructures used in tissue engineering, nanofibers are very attractive for biomedical applications, as they feature a fibrous structure similar to that of the natural ECM and possess an extremely high surface-to-volume ratio. In addition, nanofibers can be organized and adapted into a wide variety of scaffold sizes and shapes [23, 34]. To produce such fibers, PCL was selected as the base material due its slow biodegradation, low cost, simple processing, biocompatibility, and bioresorption rate appropriate for bone tissue regeneration. Moreover, the PCL polymer is approved by the Food and Drug Administration (FDA) for biomedical applications [35–37]. Several studies reported successful use of PCL scaffolds for tissue regeneration purposes. Diba et al. presented a novel forsterite/PCL nanocomposite porous scaffold, which featured porosity and pore interconnectivity suitable for bone tissue engineering applications [38]. Kamath et al. engineered a 3D porous PCL scaffold impregnated with resveratrol-loaded albumin nanoparticles (RNP). The controlled and prolonged release of resveratrol significantly improved mineralization, which can be of significant therapeutic value in bone tissue engineering processes [39]. He et al. showed that electrospun gelatin/PCL membranes embedded with bone marrow-derived stem cells/chondrocyte coculture facilitated the formation of high-quality and well-distributed neocartilage using strategy, indicating its suitability in stem cell-based cartilage engineering [40]. Luo et al. have reported on the unique nanotopographical effect of electrospun PCL nanofibrous mesh on the extent of foreign body reactions in a tissue engineering chamber, which led to reduced capsule formation and a larger volume of adipose flap [41]. Lastly, Eftekhari et al. also reported on a larger quantity of newly formed lamellar bone in healing rabbit femoral defects 45 days after treatment with a nanocomposite PCL versus a hydroxyapatite scaffold [42].

Although extensive research has established PCL as preferred polymer material for tissue engineering purposes, PCL scaffolds still do not exhibit the mechanical properties and bioactive behavior for cell proliferation and differentiation [35]. Its intrinsic hydrophobic nature also limits surface wetting and interaction with biological fluids, both of which are required for cell adhesion and proliferation. These drawbacks can be overcome by combining PCL matrix with other bioactive phases of biomaterials, such as hydroxyapatite or bioglass [36, 37, 43]. In this study, scaffold surface properties were modified by integrating Pro Osteon particles composed of hydroxyapatite, which is a bioresorbable ceramic with a chemical, biological, and crystal makeup similar to that of native apatite in the human skeleton. Its properties allow it to chemically bond with living bone tissue and its presence was expected to enhance the osteoconductivity and porosity of the scaffold [8]. Hence, the 3D hybrid construct combines the benefits of the two materials, namely, the strength and slow biodegradability of PCL and the osteoconductivity, bioactivity, and biocompatibility of hydroxyapatite that enables cell adhesion and proliferation [8, 36, 44].

To evaluate the biocompatibility and functionality of the hybrid scaffold, adipose tissue-derived MSCs were embedded and monitored over time. This cell type was selected due to its accessibility, expansibility, and capacities to both self-renew and differentiate into numerous different tissue types [12]. When compared with bone marrow-derived stem cells, adipose-derived stem cells (ASCs) can be more readily and safely harvested, in relative abundance, by modern liposuction techniques [45]. Furthermore, unlike the traditional isolation method described by Locke et al. [45], the method used in this study is rapid and efficient, which relies on ASC adherence to plastic [13]. The mesenchymal origin of the isolated ASCs was verified by their spindle-like morphology, their high expression of mesenchyme-specific markers CD73, CD90, and CD105, and their negative expression of hematopoietic and the leukocyte markers CD34, CD45, CD11b, and HLA-DR [12]. Furthermore, the osteogenic potential of the isolated cells was shown by positive staining for ALP and with the Alizarin Red Stain after 28 days in osteogenic conditions, which is in accordance with a report by David et al. [46]. In conclusion, the isolated cells were verified as classic mesenchymal stem cells with strong osteogenic potential.

The capability of the hybrid scaffold to retain the MSCs and to support their growth* in vitro* and* in vivo* was demonstrated by increased proliferation rates* in vitro*, as was shown using the Alamar Blue assay. SEM and histological analysis of the cell-embedded scaffold further confirmed infiltration of the cells into the scaffold and their expansion onto the PCL fibers. These results provide proof of the low toxicity of the PCL polymer and confirm that the engineered composite scaffold is biocompatible and supports growth of human ASCs.

Furthermore, within 8 weeks of implanting the scaffold in an animal model, the seeded cells began to differentiate into a variety of cell types and form several tissue types within the implanted scaffold area. Moreover, the osteogenic capacity of the cell-embedded scaffold was demonstrated by formation of new bone tissue without any growth factor stimulation. Although the hybrid scaffold was fabricated to be bone-specific scaffold, other tissues have formed within this scaffold. Such tissue formation may be due to various reasons; first, the cells were cultured in inductive medium prior to seeding onto scaffold, which triggers their differentiation, but their commitment to osteoblast fate was not really fulfilled. In order to determine the fate of these cells, specific triggers should be used like adding growths factors (BMP-2) to the seeded scaffold or culturing the cells prior to seeding into osteoinductive culture media that trigger differentiation of osteoblast cells. Secondly, in order to implant scaffolds subcutaneously, surgical incision into the dermal layers of the animal model was made. Producing this incision during the surgical process triggers cells to secrete growth factors needed for repairing tissues, especially the dermal layers. Therefore, these growth factors may trigger the cells to differentiate into other type of tissues, other than bone tissue. In order to make our hybrid scaffold more specified for bone formation, osteogenic differentiation should be induced, which was demonstrated to be the basis of bone regeneration [47]. Yet, the MSC-embedded hybrid scaffolds failed to demonstrate superiority in bone formation over Pro Osteon implanted alone. This insignificant difference in bone formation could be due to the fact that both the hybrid scaffold and Pro Osteon particles have the same osteoconductive material similar to previous results where bone formation depends on the osteoconductive material type and quantity [16]. However, the hybrid scaffold surpasses Pro Osteon in its 3D architecture that combines the nanofibers and osteoconductive material, mimicking the nano- and microstructure of bone ECM. Its structure facilitates transplanting procedure as a one matrix, unlike the unattached Pro Osteon particles; therefore it will be greatly preferred for clinical applications, especially in cases of large bone loss. The structure and composition of such engineered scaffold could be further tuned and modified by changing the used polymers/biomaterials or by embedding growth factors to adjust for the tissue regeneration.

In conclusion, the presented results demonstrate the biocompatibility and functionality of an MSC-embedded hybrid PCL-hydroxyapatite scaffold in the host and show promising potential for bone tissue engineering applications. Future studies must consider integration of growth factor stimulation to boost differentiation of osteogenic cells and tissue prevascularization to supply oxygen and nutrients and to clear metabolic byproducts from the growing tissue [48–51]. Future efforts should focus on developing a vascularized hybrid PCL-hydroxyapatite electrospun scaffold.

## References

[1] Dimitriou R., Jones E., McGonagle D., Giannoudis P. V. (2011). Bone regeneration: current concepts and future directions. *BMC Medicine*.

[2] Mata A., Geng Y., Henrikson K. J. (2010). Bone regeneration mediated by biomimetic mineralization of a nanofiber matrix. *Biomaterials*.

[3] Amini A. R., Laurencin C. T., Nukavarapu S. P. (2012). Bone tissue engineering: recent advances and challenges. *Critical Reviews in Biomedical Engineering*.

[4] Rose F. R. A. J., Oreffo R. O. C. (2002). Bone tissue engineering: hope vs hype. *Biochemical and Biophysical Research Communications*.

[5] Stevens M. M. (2008). Biomaterials for bone tissue engineering. *Materials Today*.

[6] Leong N. L., Jiang J., Lu H. H. Polymer-Ceramic Composite Scaffold Induces Osteogenic Differentiation of Human Mesenchymal Stem Cells.

[7] Khan Y., Yaszemski M. J., Mikos A. G., Laurencin C. T. (2008). Tissue engineering of bone: material and matrix considerations. *The Journal of Bone & Joint Surgery*.

[8] Oyefusi A., Olanipekun O., Neelgund G. M. (2014). Hydroxyapatite grafted carbon nanotubes and graphene nanosheets: Promising bone implant materials. *Spectrochimica Acta Part A: Molecular and Biomolecular Spectroscopy*.

[9] Liu X., Ma P. X. (2004). Polymeric scaffolds for bone tissue engineering. *Annals of Biomedical Engineering*.

[10] Birmingham E., Niebur G. L., Mchugh P. E., Shaw G., Barry F. P., McNamara L. M. (2012). Osteogenic differentiation of mesenchymal stem cells is regulated by osteocyte and osteoblast cells in a simplified bone niche. *European Cells and Materials*.

[11] Rosenbaum A. J., Grande D. A., Dines J. S. (2008). The use of mesenchymal stem cells in tissue engineering: a global assessment. *Organogenesis*.

[12] Docheva D., Popov C., Mutschler W., Schieker M. (2007). Human mesenchymal stem cells in contact with their environment: surface characteristics and the integrin system. *Journal of Cellular and Molecular Medicine*.

[13] Zeng G., Lai K., Li J. (2014). A rapid and efficient method for primary culture of human adipose-derived stem cells. *Organogenesis*.

[14] Nseir N., Regev O., Kaully T., Blumenthal J., Levenberg S., Zussman E. (2013). Biodegradable scaffold fabricated of electrospun albumin fibers: Mechanical and biological characterization. *Tissue Engineering - Part C: Methods*.

[15] Srouji S., Kizhner T., Ben David D., Riminucci M., Bianco P., Livne E. (2009). The schneiderian membrane contains osteoprogenitor cells: in vivo and in vitro study. *Calcified Tissue International*.

[16] Srouji S., Ben-David D., Funari A., Riminucci M., Bianco P. (2013). Evaluation of the osteoconductive potential of bone substitutes embedded with schneiderian membrane- or maxillary bone marrow-derived osteoprogenitor cells. *Clinical Oral Implants Research*.

[17] Orciani M., Fini M., Di Primio R., Mattioli-Belmonte M. (2017). Biofabrication and Bone Tissue Regeneration: Cell Source, Approaches, and Challenges. *Frontiers in Bioengineering and Biotechnology*.

[18] Lavik E., Langer R. (2004). Tissue engineering: Current state and perspectives. *Applied Microbiology and Biotechnology*.

[19] Srouji S., Kizhner T., Suss-Tobi E., Livne E., Zussman E. (2008). 3-D Nanofibrous electrospun multilayered construct is an alternative ECM mimicking scaffold. *Journal of Materials Science: Materials in Medicine*.

[20] John Martin T., Kong Wah Ng, Nicholson G. C. (1988). 1 Cell biology of bone. *Best Practice & Research Clinical Endocrinology & Metabolism*.

[21] Bajaj P., Schweller R. M., Khademhosseini A., West J. L., Bashir R. (2014). 3D biofabrication strategies for tissue engineering and regenerative medicine. *Annual Review of Biomedical Engineering*.

[22] Puppi D., Zhang X., Yang L., Chiellini F., Sun X., Chiellini E. (2014). Nano/microfibrous polymeric constructs loaded with bioactive agents and designed for tissue engineering applications: a review. *Journal of Biomedical Materials Research Part B: Applied Biomaterials*.

[23] Liu H., Ding X., Zhou G., Li P., Wei X., Fan Y. (2013). Electrospinning of nanofibers for tissue engineering applications. *Journal of Nanomaterials*.

[24] Annabi N., Nichol J. W., Zhong X. (2010). Controlling the porosity and microarchitecture of hydrogels for tissue engineering. *Tissue Engineering - Part B: Reviews*.

[25] Yang S., Leong K.-F., Du Z., Chua C.-K. (2001). The design of scaffolds for use in tissue engineering. Part I. Traditional factors. *Tissue Engineering Part A*.

[26] Srouji S., Kizhner T., Livne E. (2006). 3D scaffolds for bone marrow stem cell support in bone repair.. *Journal of Regenerative Medicine*.

[27] Huang Z.-M., Zhang Y. Z., Kotaki M., Ramakrishna S. (2003). A review on polymer nanofibers by electrospinning and their applications in nanocomposites. *Composites Science and Technology*.

[28] Mitsak A. G., Kemppainen J. M., Harris M. T., Hollister S. J. (2011). Effect of polycaprolactone scaffold permeability on bone regeneration in vivo. *Tissue Engineering Part A*.

[29] Hollister S. J. (2005). Porous scaffold design for tissue engineering. *Nature Materials*.

[30] Narayanan G., Gupta B. S., Tonelli A. E. (2015). Estimation of the poly (*ε*-caprolactone) [PCL] and *α*-cyclodextrin [*α*-CD] stoichiometric ratios in their inclusion complexes [ICs], and evaluation of porosity and fiber alignment in PCL nanofibers containing these ICs. *Data in Brief*.

[31] Daish C., Blanchard R., Gulati K. (2017). Estimation of anisotropic permeability in trabecular bone based on microCT imaging and pore-scale fluid dynamics simulations. *Bone Reports*.

[32] Karande T. S., Ong J. L., Agrawal C. M. (2004). Diffusion in musculoskeletal tissue engineering scaffolds: design issues related to porosity, permeability, architecture, and nutrient mixing. *Annals of Biomedical Engineering*.

[33] Ochoa I., Sanz-Herrera J. A., García-Aznar J. M., Doblaré M., Yunos D. M., Boccaccini A. R. (2009). Permeability evaluation of 45S5 Bioglass-based scaffolds for bone tissue engineering. *Journal of Biomechanics*.

[34] Leung V., Ko F. (2011). Biomedical applications of nanofibers. *Polymers for Advanced Technologies*.

[35] Woodruff M. A., Hutmacher D. W. (2010). The return of a forgotten polymer—polycaprolactone in the 21st century. *Progress in Polymer Science*.

[36] Hassan M. I., Sultana N., Hamdan S. (2014). Bioactivity assessment of poly(*ε* -caprolactone)/hydroxyapatite electrospun fibers for bone tissue engineering application. *Journal of Nanomaterials*.

[37] Diba M., Kharaziha M., Fathi M. H., Gholipourmalekabadi M., Samadikuchaksaraei A. (2012). Preparation and characterization of polycaprolactone/forsterite nanocomposite porous scaffolds designed for bone tissue regeneration. *Composites Science and Technology*.

[38] Diba M., Fathi M. H., Kharaziha M. (2011). Novel forsterite/polycaprolactone nanocomposite scaffold for tissue engineering applications. *Materials Letters*.

[39] Kamath M. S., Ahmed S. S. S. J., Dhanasekaran M., Winkins Santosh S. (2013). Polycaprolactone scaffold engineered for sustained release of resveratrol: therapeutic enhancement in bone tissue engineering. *International Journal of Nanomedicine*.

[40] He X., Feng B., Huang C. (2015). Electrospun gelatin/polycaprolactone nanofibrous membranes combined with a coculture of bone marrow stromal cells and chondrocytes for cartilage engineering. *International Journal of Nanomedicine*.

[41] Luo L., He Y., Chang Q. (2016). Polycaprolactone nanofibrous mesh reduces foreign body reaction and induces adipose flap expansion in tissue engineering chamber. *International Journal of Nanomedicine*.

[42] Eftekhari H., Jahandideh A., Asghari A., Akbarzadeh A., Hesaraki S. (2017). Assessment of polycaprolacton (PCL) nanocomposite scaffold compared with hydroxyapatite (HA) on healing of segmental femur bone defect in rabbits. *Artificial Cells, Nanomedicine and Biotechnology*.

[43] Patrício T., Domingos M., Gloria A., Bártolo P. (2013). Characterisation of PCL and PCL/PLA scaffolds for tissue engineering. *Procedia CIRP*.

[44] Wutticharoenmongkol P., Pavasant P., Supaphol P. (2007). Osteoblastic phenotype expression of MC3T3-E1 cultured on electrospun polycaprolactone fiber mats filled with hydroxyapatite nanoparticles. *Biomacromolecules*.

[45] Locke M., Windsor J., Dunbar P. R. (2009). Human adipose-derived stem cells: isolation, characterization and applications in surgery. *ANZ Journal of Surgery*.

[46] David D. B., Reznick A. Z., Srouji S., Livne E. (2008). Exposure to pro-inflammatory cytokines upregulates MMP-9 synthesis by mesenchymal stem cells-derived osteoprogenitors. *Histochemistry and Cell Biology*.

[47] Hayrapetyan A., Jansen J. A., Van Den Beucken J. J. J. P. (2015). Signaling pathways involved in osteogenesis and their application for bone regenerative medicine. *Tissue Engineering - Part B: Reviews*.

[48] Santos M. I., Reis R. L. (2010). Vascularization in bone tissue engineering: physiology, current strategies, major hurdles and future challenges. *Macromolecular Bioscience*.

[49] Laschke M. W., Menger M. D. (2012). Vascularization in tissue engineering: angiogenesis versus inosculation. *European Surgical Research*.

[50] Srouji S., Ben-David D., Lotan R., Livne E., Avrahami R., Zussman E. (2011). Slow-release human recombinant bone morphogenetic protein-2 embedded within electrospun scaffolds for regeneration of bone defect: In vitro and in vivo evaluation. *Tissue Engineering Part: A*.

[51] Holmes B., Castro N. J., Zhang L. G., Zussman E. (2012). Electrospun fibrous scaffolds for bone and cartilage tissue generation: Recent progress and future developments. *Tissue Engineering - Part B: Reviews*.

